# Studying Public Perception about Vaccination: A Sentiment Analysis of Tweets

**DOI:** 10.3390/ijerph17103464

**Published:** 2020-05-15

**Authors:** Viju Raghupathi, Jie Ren, Wullianallur Raghupathi

**Affiliations:** 1 Koppelman School of Business, Brooklyn College of the City University of New York, Brooklyn, NY 11210, USA; VRaghupathi@brooklyn.cuny.edu; 2 Gabelli School of Business, Fordham University, New York, NY 10023, USA; JRen11@fordham.edu

**Keywords:** healthcare, measles, sentiment analysis, text analysis, vaccination, vaccine, social media, Twitter

## Abstract

Text analysis has been used by scholars to research attitudes toward vaccination and is particularly timely due to the rise of medical misinformation via social media. This study uses a sample of 9581 vaccine-related tweets in the period 1 January 2019 to 5 April 2019. The time period is of the essence because during this time, a measles outbreak was prevalent throughout the United States and a public debate was raging. Sentiment analysis is applied to the sample, clustering the data into topics using the term frequency–inverse document frequency (TF-IDF) technique. The analyses suggest that most (about 77%) of the tweets focused on the search for new/better vaccines for diseases such as the Ebola virus, human papillomavirus (HPV), and the flu. Of the remainder, about half concerned the recent measles outbreak in the United States, and about half were part of ongoing debates between supporters and opponents of vaccination against measles in particular. While these numbers currently suggest a relatively small role for vaccine misinformation, the concept of herd immunity puts that role in context. Nevertheless, going forward, health experts should consider the potential for the increasing spread of falsehoods that may get firmly entrenched in the public mind.

## 1. Introduction

In this research, we use Twitter data to discover and describe public sentiment regarding vaccination. This is timely since there was a large measles outbreak in the United States and other parts of the world in 2019 [1,2,3]. Additionally, approximately 142,000 people are estimated to have died from measles in 2017 [4]. This could have been prevented with timely vaccination [5]. By understanding the public sentiment about vaccination, government officials and public health policy makers can design more effective communication, education and policy implementation strategies to reach out to the public [6,7,8]. We explore the following research question in this study: what is the public perception regarding vaccination?

We apply text analysis techniques including word clouds and sentiment analysis on the corpus of tweets from Twitter as a representative of social media [8]. Data were clustered into topics using the term frequency–inverse document frequency (TF-IDF) technique.

The rest of the paper is organized as follows: Section 2 introduces key background information for the research and reviews previous literature; Section 3 discusses the methodology; Section 4 describes the results with a discussion; Section 5 provides the scope and limitations of the research; Section 6 provides an integrative discussion; and finally, Section 7 draws conclusions and discusses implications.

## 2. Background

The outbreak of infectious diseases, and the potential for vaccination to prevent them, is a rich topic in the study of public opinion and sentiment. There are various social media outlets (e.g., Twitter, blogs, Facebook, Snapchat, etc.) for the public to discuss and disseminate their opinions and experiences. Along these lines, we selected a corpus of text related to vaccine-related comments on Twitter, as a representative social media channel. We selected Twitter since it focuses on key words and allows people to post to a wider audience than other channels. Moreover, Twitter has been successfully used in prior research on blogs, images and textual information [9,10,11,12,13,14,15]. While we focused on tweets related to vaccines—and not necessarily to measles-related vaccines—it so happened that since the timeframe of our research coincided with the measles outbreak in the United States, most of the tweets naturally related to it. Therefore, in this section, we first lay the foundation for our research with a discussion on the incidence of measles and the status of vaccination for the same; we then cover the general topic of sentiment towards vaccination; next, we describe the dissemination of such sentiments through the channel of social media, highlighting the relevant paradox of information and misinformation; and finally we outline the potential and appropriateness of sentiment analysis as a technique for our research objective of analyzing public perception in healthcare.

### 2.1. Increasing Incidence of Measles and Low Vaccination Rates

The outbreak of measles as a viral infection is serious since it is especially dangerous for small children [4,16]. It can cause high fever and a spotty rash over the body. Prior to 1963, measles was responsible for about 400–500 deaths each year [17]. The measles vaccine, which was introduced after 1963, caused measles to be classified as a vaccine-preventable disease throughout the world [4,5,16]. Measles was virtually eliminated in the United States by the year 2000 [18]. The impact of measles in the post-elimination period is evaluated on the basis of the immunological cost, financial cost and the resulting strain on the healthcare system [19]. Unlike other diseases, measles causes post-infection immunosuppression, often making the person susceptible to contracting other bacterial and viral infections for a period of three years after the onset of measles. Additionally, this immunosuppression may also affect an individual’s immunological response to other vaccines [19]. Therefore, the risk of mortality and morbidity is escalated. The financial cost of measles includes not just a direct treatment-related costs but also quarantine-related costs. There is a lot of strain on the healthcare system since a response to measles requires the diversion of human resources from other programs and functions. Considered together, these three factors highlight the importance of preventing the spread of measles in an economy.

In this aspect, the Centers for Disease Control and Prevention (CDC) proposes that measles is easily preventable by vaccination [17,20]. A person who carries the virus can spread it through talk, touch and cough. If unvaccinated people are in a room with a person who carries the measles virus (even if that person has left the room), the unvaccinated people in the room have high probability of catching droplets in the air. As a result, those individuals can get infected [21].

**Figure 1:**
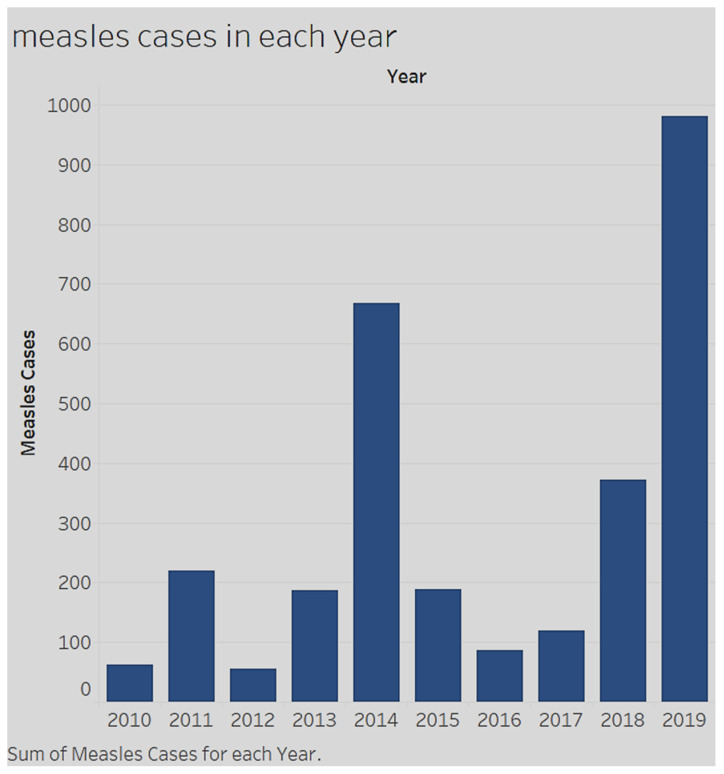
Measles cases in the United States (2010–2019) (https://www.cdc.gov/measles/cases-outbreaks.html).

There has been a marked increase in the number of measles cases in the United States over the last 10 years. Figure 1 shows the number of measles cases from 2010 to 2019. From 2000 to 2009, fewer than 100 Americans were infected with measles each year. This number grew to 981 cases in May 2019 [22]. According to the CDC the number of patients diagnosed with measles in the U.S. continues to grow [22]. In the first five months of 2019, 981 individual cases of measles were confirmed in 26 states in the U.S [22,23]. Unfortunately, this number continued to grow through August 2019. In Figure 1, the number of cases over a five-month period was higher than in previous years from 2010 to 2018. Moreover, this is the most significant number in the U.S. since 2002. The number of reported cases grew to 1215 by 22 August 2019 [24].

There is heated discussion regarding the 2019 outbreak, especially in the United States [25,26].

Travel provides opportunities for an increase in cases. In the past two years, the number of measles cases increased sharply [25,27]. In 2018, the number increased by 300% globally. According to the World Health Organization (WHO), the countries most affected include Philippines, Madagascar, India, Pakistan, Ukraine, Brazil and Yemen. Travelers from these hotspot countries may carry the virus to the U.S, where its spread is related to vaccination rates [27]. Religious belief against vaccination for measles prevention is another factor in the outbreak [28,29,30]. Statistics show geographic clusters of the virus. In fact, the resurgence of measles is not widespread across the U.S. but occurs in local areas [31,32,33,34]. Seventy-five percent of recent measles cases occurred in close-knit communities in the states of California, Washington, New Jersey and New York [19,34,35,36]. Moreover, most of these cases (over 600) were localized in orthodox Jewish communities in New York City and its suburb, Rockland. In these communities, people share spaces at school and worship, creating ideal conditions for the virus to spread [19,37].

Another reason for a low vaccination rate is the public’s lack of trust in government. A recent example from China shows how a vaccine scandal can shake the public’s trust in government and reduce vaccination rates [38,39,40]. Vaccines have a booming market in China since pharmaceutical innovation is encouraged and prioritized by the government. The industry produces more than $3 billion in revenue per year [41,42,43]. However, there are also issues concerning the violation of standards. For example, in one of three Chinese vaccine crises since 2010, Changchun Changsheng, one of the largest drug producers in China, violated standards in the manufacturing of at least 250,000 doses of a vaccine for diphtheria, tetanus and whooping cough. A series of recent vaccine scandals has deepened skepticism about the Chinese government’s response; many parents in China have lost faith in vaccines produced in their own country [44,45]. Simultaneously and comparably in the U.S., regardless of the reasons for the recurrence of the virus in the U.S., the most critical issue affecting its spread is the rate of vaccination in the population [46,47,48].

### 2.2. Sentiments towards Vaccination

A vaccine is a biological preparation that provides active acquired immunity to a disease [49,50,51]. It typically contains an agent which resembles a specific disease-causing microorganism. The agent is derived from weak or dead forms of the microbe, its toxins or one of its surface proteins [49,50,51]. The human body identifies the agent as a threat and stimulates the immune system to produce antibodies to destroy it [49]. Vaccines work because those antibodies will recognize and destroy the actual disease-causing microorganism if they encounter it in the future [49,50,51]. Evidence indicates that vaccines are a safe and effective approach to protecting individual and public health [49,50,51]. However, for a vaccine to work effectively at the population level, a good percentage of a community needs to get immunized [52,53,54] to achieve so-called “herd immunity” [55,56,57]. Herd immunity can prevent disease from spreading through populations even when some individuals remain unvaccinated [55,56,57]. Thus, a mostly vaccinated population protects those of its members who cannot be vaccinated, such as newborns, people with vaccine allergies and cancer patients [55,56,57,58,59].

However, vaccination rates (for diseases such as measles) in the U.S. (e.g., Connecticut) have continued to fall [24]. In fact, an antivaccine sentiment has been building in the U.S. for decades [60,61,62]. In 2019, the debate over the vaccination requirement in schools was reignited [63,64,65]. A majority of measles cases reported since the beginning of the outbreak in October 2018 involved unvaccinated children in Hasidic Jewish communities [66,67,68]. In response to the measles epidemic, in June 2019, New York state lawmakers approved a new law that banned religious exemptions to school vaccination requirements [69]. This law was opposed by thousands of anti-vaccination parents [70].

In addition to attitudes towards vaccination, the spread of misinformation is another prime factor in the resurgence of measles [71]. The anti-vaccination movement, led by a fringe group of parents who oppose the vaccine, believe that, contrary to scientific studies, the vaccine’s chemical makeup can cause autism [72]. It is easy for misinformation to be spread through social media or by word of mouth. A critical factor that contributed to the widespread dissemination of “fake news” was the publication of an article by a gastroenterologist Andrew Wakefield in the medical journal *Lancet* in 1988 [73,74,75]. The publication discussed 12 children who had pervasive developmental disorders associated with gastrointestinal symptoms [73,74,75]. According to retrospective accounts by their parents or physicians, eight of the children also had behavioral issues associated with the measles, mumps and rubella (MMR) vaccination [76]. Dr. Wakefield’s study has since been retracted because it was proven to be the result of serious professional misconduct, and most of the children in his original study were revealed to have symptoms that started either before, or much after, the MMR vaccination [73,74,75]. In addition to the problems with the original study, Dr. Wakefield was found to be engaging in unnecessary, undesirable and harmful procedures on children without any approval from the hospital ethics committee [73,74,75]. Moreover, he was found to be soliciting financial donations from the Legal Aid Board—a group pursuing legal action for children who were alleged to be vaccine-damaged [73,77,78,79,80,81]. In addition, on behalf of parents, he promoted vaccines to rival the established MMR (measles, mumps, and rubella) vaccine. The British General Medical Council finally revoked Dr. Wakefield’s medical license in the U.K in 2010. Even through the fraudulent results of the research have all been refuted by the scientific community [73,74,75], the study nevertheless has maintained its influence in social media [82,83]. Additionally, celebrities with “vaccine hesitancy” also got involved, sharing their opinions with their active audiences. This type of movement can be critical, especially when using social networking systems [84]. As a result, the antivaccination sentiment is a byproduct of misinformation on the Internet and remains a driving factor for reduced vaccination rates [85,86].

### 2.3. Social Media and the Dissemination of (Mis)information

The domain of healthcare, specifically, is characterized by high levels of public concern and therefore, misinformation can spread rampantly via social media. Erroneous information (or misinformation) related to vaccines and vaccine safety has been problematic for many years [87].

On one hand, great progress has been made in national immunization programs thanks to effective vaccines for children. In addition, there is general societal consensus about the rare side effects of vaccines and the view of vaccines as a public good. Throughout the 20th century, the Food and Drug Administration’s (FDA) Center for Biologics Evaluation and Research (CBER) has been instrumental in safeguarding scientific discoveries such as vaccines. Furthermore, the vaccine industry became the most closely regulated industry in the U.S. because of severe vaccine misadventures in the 20th century [88]. On the other hand, immunization programs in the U.S and in Europe (U.K, Netherlands, Germany and Switzerland) [89,90,91,92] have dealt with many challenges, the predominant one being the dissemination of misinformation on vaccine safety [93,94]. In this regard, there is really very limited evidence-based information to support ideas related to the dangers of vaccines. Therefore, antivaccination activists have typically relied on the power of experience (or anecdotes) to influence large groups of parents with doubts and fears [5,86], through discussions on the Web and social media outlets [95]. In these arenas, accounts of perceived vaccine injury, together with access to Dr. Wakefield’s now-retracted research study [75,86], have resulted in a large amount of vaccine hesitancy in parents, including vaccine refusal and delayed vaccine schedules [95]. Added to this is the unregulated nature of the Internet in giving people unlimited freedom of information content and disclosure [85,96]. Researchers have explored online anti-vaccination misinformation by analyzing arguments on anti-vaccination websites, studying the levels of misinformation, and examining discourses to support antivaccination claims [85]. Some studies pointed out that antivaccination websites have high ranks in search engines [97,98]. Moreover, some studies show that people who have strong negative attitudes and opinions are more active in posting information on social media and therefore exert a strong influence on people’s attitudes [85,98,99]. In recent years, social media has become a prime channel for distributed global communication. As the demand for transparency in the experiences from vaccination had increased, social media has evolved to become a platform not only for patient engagement but also for empowerment. Social networks play a huge role in modulating individual health behaviors [100,101]. Twitter, for one, has been a platform for individuals to show their utterances to the public. Currently, 21% of U.S. adults use Twitter; forty-two percent of those users visit the Twitter platform on a daily basis [102]. Currently, there are over 69 million monthly active Twitter users in the U.S. [9]. Formal evaluations of vaccine studies can benefit from incorporating social media discussions of vaccine users as complementary evaluations [10]. As a result, there is rich potential for extracting health information and studying the dynamics of health behaviors on Twitter [103].

### 2.4. Sentiment Analysis

To this effect, sentiment analysis has been deployed as a natural language processing task at varying degrees of granularity. Natural language processing began with performing classification tasks on a document level [104,105]. Initially, analysis was handled at the sentence level [106,107], and later progressed to a phrase level [108]. In recent years, Agarwal et al. [109] brought natural language processing to a new level, using lexical scoring with n-gram analysis to model the effect of context in order to perform phrase-level polarity and n-gram analysis. Considering the advances in natural language processing and text analytics, sentiment analysis has increasingly become a beneficial tool to explore individuals’ attitude patterns toward vaccines and vaccination in particular, and public health in general [109]. Several studies have applied sentiment analysis to explore sentiment trends about vaccination through social media like Twitter. For example, Du et al. [110] applied machine learning-based approaches to examine sentiment trends using Twitter data [110]. Surian et al. conducted topic modeling and community detection to identify negative sentiments about Human Papillomavirus (HPV) vaccines on Twitter [11]. Salathé and Khandelwal [8] defined a score for sentiment on the H1N1 vaccination. Radzikowski et al. [12] applied a quantitative analysis to determine a measles vaccination narrative on Twitter [12].

This article augments this literature by employing the natural language toolkit (NLTK) to perform sentiment analysis to explore patterns and trends among collected Twitter data to understand the opinions of people towards measles vaccination. Insights from the study help formulate and shape public policy with regard to prevention of measles.

## 3. Methods

In this article, a corpus of vaccine-related text messages on Twitter (tweets) is examined. We selected Twitter as our social media platform since it focuses on key words and allows posting to a wider audience when compared to other platforms like Facebook. We did a random selection of six tweets as a pilot, in order to get initial insight into the various opinions on vaccines. We performed several analyses on vaccine-related tweets, including word count frequency, word cloud generation, word co-occurrence, TF-IDF clustering and sentiment scoring of tweets [111,112]. The TF-IDF technique searches through a corpus of documents to determine key words. The text data are then transformed into a TF-IDF matrix to provide the frequency of a word in the corpus. Collectively, these text analyses have been demonstrated to be comparable to traditional survey analytical methods in describing individual opinions on a vaccine [6,7,8,13,14,113,114]. Evaluation of research adopting sentiment analyses for vaccine-related tweets have created an awareness of the need for disseminating vaccine-related information.

This study uses a sample of 9581 vaccine-related tweets in the period 1 January 2019 to 5 April 2019. This time period is of the essence because during this time a measles outbreak was prevalent in the United States and public debate on vaccination was raging. We therefore decided to zoom in and capture public perception during this peak period. All the tweets with the keyword “vaccine” were scraped globally. Only tweets in English were included. They were coded in Python programming language to conduct vaccine-related sentiment analysis. The text data were put into data frames using Pandas packages in Python. Pandas package is an open source library that provides easy-to-use, high performance data structures and data analysis tools for Python language. The NLTK (Natural Language ToolKit), which is a suite of libraries and programs for natural language processing written in the Python programming language was used. In the next step, three stemmers (WordNet, Lancaster Stemmer and Porter Stemmer) in the NLTK were used to prune the text data. The three stemmers provided a comparison for improved performance. The Valence Aware Dictionary and Sentiment Reasoner (VADER) was used to handle sentiment analysis on the text data. In sentiment analysis, each sentence is analyzed to determine a sentiment score based on whether it conveys a positive, negative or neutral sentiment. VADER is based on lexicons of sentiment-related words. Each word will have a sentiment rating—for example a positive word (e.g., “good”) has a positive sentiment rating of 1.9. Words that are more positive words will have a higher sentiment rating (e.g., the word “great” will have a higher rating than “good”). The same rule applies to words that convey negative sentiments. The outcome metric has four parts: positive sentiment score; neutral sentiment score; negative sentiment score; and compound score. The compound score is the sum of the lexicon ratings, which are standardized values between −1 and 1. The compound score was used as a classifier. A tweet with a compound score more than or equal to 0.05 is identified as a positive sentiment tweet. A tweet with a compound score less than or equal to −0.05 is identified as a negative sentiment tweet. Other tweets are identified as neutral sentiment tweets. In the final step, K-means was used to perform clustering on the text data. Skikit-learn, a software machine learning library for the Python programming language, was utilized to cluster documents by topics, using a bag-of-words approach. Initially, features were extracted using the TF-IDF method since some words were more frequently used within the large text corpus (e.g., “the”, “I”, “a”, “are”). These words carried little-to-no meaningful information on the actual content of the document. If one applies the direct count data to a classifier, the frequency of these terms would overshadow the frequencies of rarer, more interesting terms. Therefore, this study aimed to reweigh the count features into the classifier to drill into the text data. As a result, TF-IDF was introduced as part of the methodology. TfidfVectorizer can convert a collection of raw documents into a matrix of TF-IDF features. Using an in-memory vocabulary to map the most frequent words to a features index, it computes a word occurrence frequency matrix. The word frequencies are reweighted using the IDF vector collected feature-wise over the corpus. Based on the TF-IDF sparse matrix, the authors used k-means for clustering. K-means is suitable for unsupervised data (or data without a label). The algorithm based on the extracted features works iteratively to assign each data point to a k-group. Overall, there were six attributes in the final dataset: (1) username; (2) content of the tweet; (3) number of replies; (4) number of retweets; (5) number of favorites; and (6) published date. The size of the final dataset was 6*9581 = 57,486. The final dataset was a comma-separated values (CSV) file. All words in the tweet content were changed to lower case. All “/n” were replaced by “‘.” The uniform resource locators (URLs) of websites in tweet content were removed because the analysis was based solely on the text data and most of the URLs were links to pictures. In the final step, the tweet content was split into individual words to transform them into vector form data that can be read by the computer.

## 4. Results

**Figure 2:**
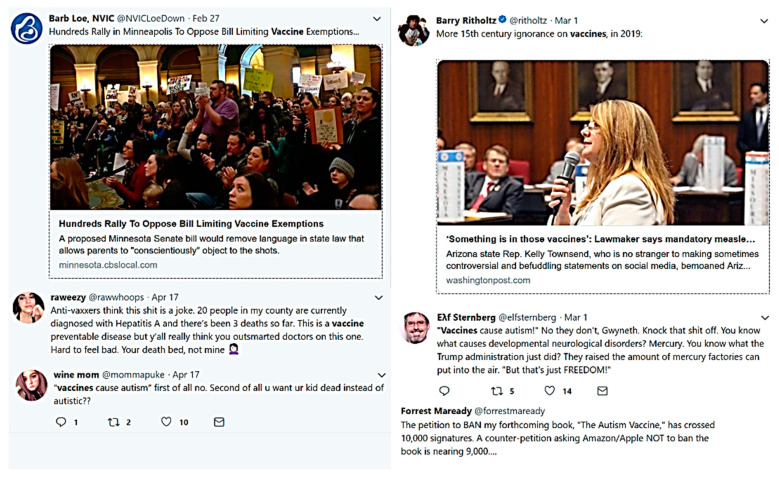
Six randomly selected tweets in the dataset.

The six randomly selected tweets provided an initial insight into the various opinions on vaccines (see Figure 2). In this figure, there is a heated debate between antivaccine activists and vaccine supporters. From the tweets, it appears that there is some public perception that vaccination is linked to autism. Three of the six tweets are skeptical as to whether vaccines cause autism. Two hold the opposite opinion, stating that “vaccines cause autism”. Another supports antivaccination. Among the other three tweets, one utterance shows that people are not really against vaccination. Two of the tweets share news about vaccines.

**Figure 3:**
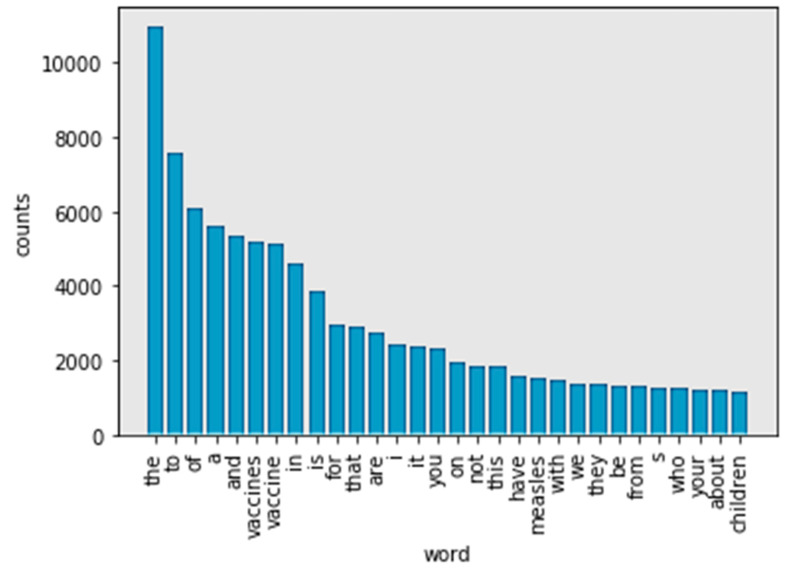
Top 30 frequency words in tokens.

In the first step, the NLTK package was utilized to tokenize content data. While text words were retained, numbers were removed. Next, a frequency dictionary was generated based on tokens. The frequency list was sorted in descending order. Figure 3 shows the top 30 frequency words in the tokens. Most of the frequency words are stop words, that is, commonly used words. Only two nouns (“vaccine” and “vaccines”) are among the top 10 keywords. With this result, there can be no insight gained from the analysis. As a result, stop words were routinely removed.

**Figure 4:**
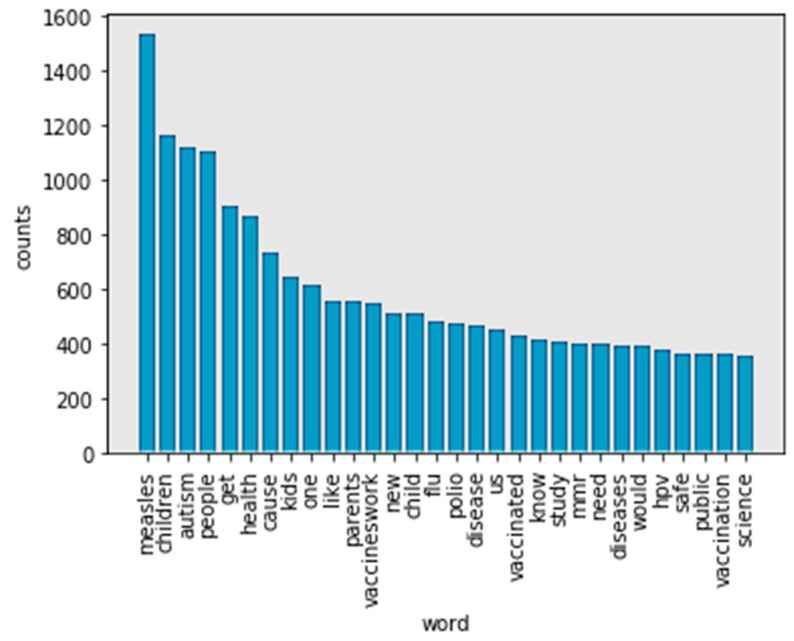
Top 30 frequency words in tokens without stop words.

The NLTK stop words list was used to identify stop words (https://nlp.stanford.edu/IR-book/html/htmledition/dropping-common-terms-stop-words-1.html). After removing the stop words, the remaining words were added to a list named “word_nostopwords”. A list was again generated. There are 30 frequency words in tokens without stop words (see Figure 4). The words “vaccine” and “vaccines” indicate the same content. In Figure 4, “measles,” “children,” and “autism” are the top three frequency words. However, words such as “kids” and “child” also frequently appear. As a result, the study found that “measles” is the most frequently used word. “Children” has the same meaning in all its appearances in the content. Therefore, these words were also stemmed.

**Figure 5:**
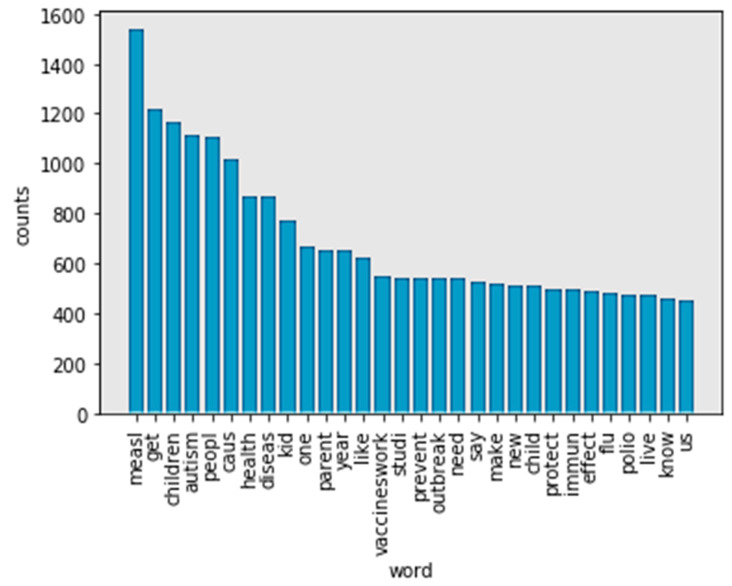
Top 30 frequency words in no-stop words tokens after processing by Porter stemmer.

Stemming refers to a simple, heuristic process that cuts off the ending of words in order to achieve a goal. Most often, this can include eliminating derivational affixes. Porter stemmer, Lancaster stemmer, and Wordnet are three techniques for stemming words. In Figure 5, the Porter stemmer did not solve the problem caused by words with a “similar” meaning. “Children” and “child” still appear separately in the frequency list.

**Figure 6:**
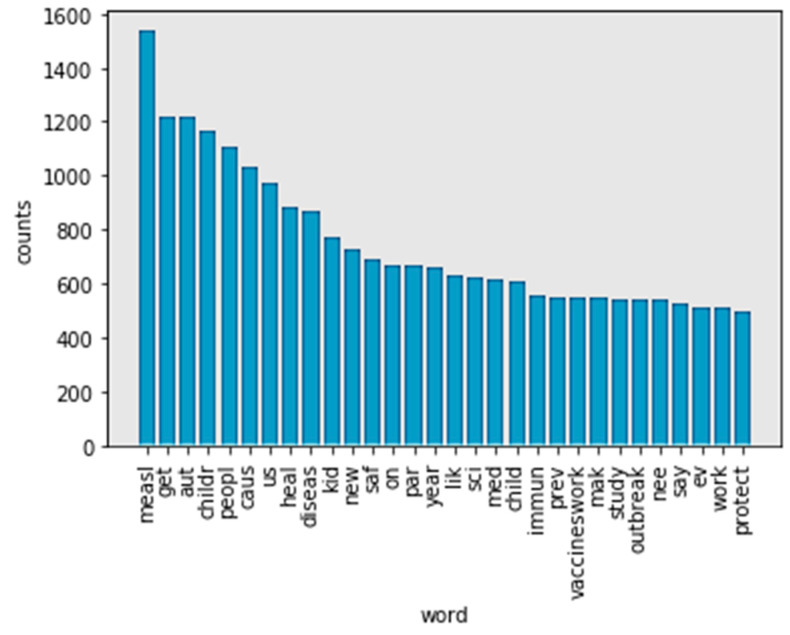
Top 30 frequency words in no-stop words tokens after processing by Lancaster stemmer.

This problem still appears in Figure 6. Using the Lancaster stemmer, the top 30 frequency words in the no-stop words tokens still show both “childr” and “child”.

**Figure 7:**
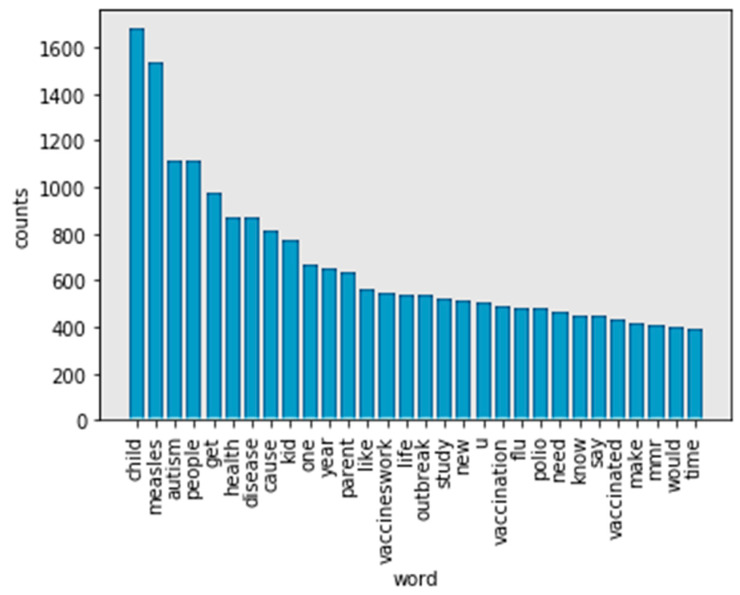
Top 30 frequency words in no-stop words tokens after processing by WordNet.

Figure 7 (WordNet) has a different most frequent word compared to Figure 5 (Porter stemmer) and Figure 6 (Lancaster stemmer). For example, “child” is the most frequent word in tokens. This is more reasonable. In comparing Figure 5 (Porter stemmer) and Figure 6 (Lancaster stemmer), there are fewer words that have overlapping meanings using WordNet as a stemmer. In this case, WordNet achieved a better performance. The top 30 words in tokens mention “measles” (1534), “autism” (1117), “flu” (482) and “polio” (472), respectively. The words “child” (1681), “people” (1110), “kid” (769), and “parent” (631) are the most-mentioned figures, respectively. “Vaccineswork” is a frequently mentioned positive sentiment word in this study (549 mentions in 9581 tweets).

**Figure 8:**
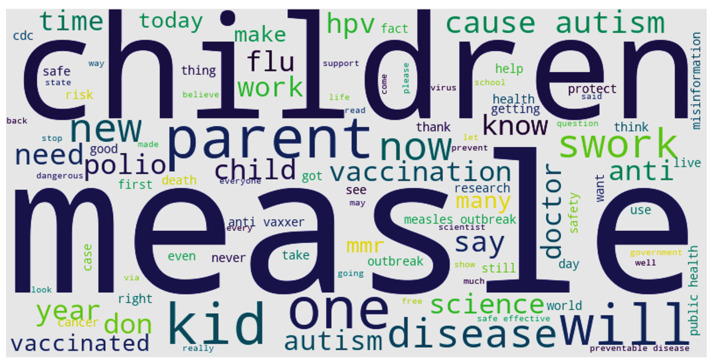
Word cloud of content.

The word cloud in Figure 8 displays the words most frequently found in the corpus of tweets. The larger the word’s size in the cloud, the more often it occurs in the corpus. The figure shows that words like “measle,” “children,” “autism,” and “parents” are distinct in the case data set. “Measle” and “autism” are related to disease. “Children” and “parents” are the entities who use Twitter to talk about vaccines.

**Figure 9:**
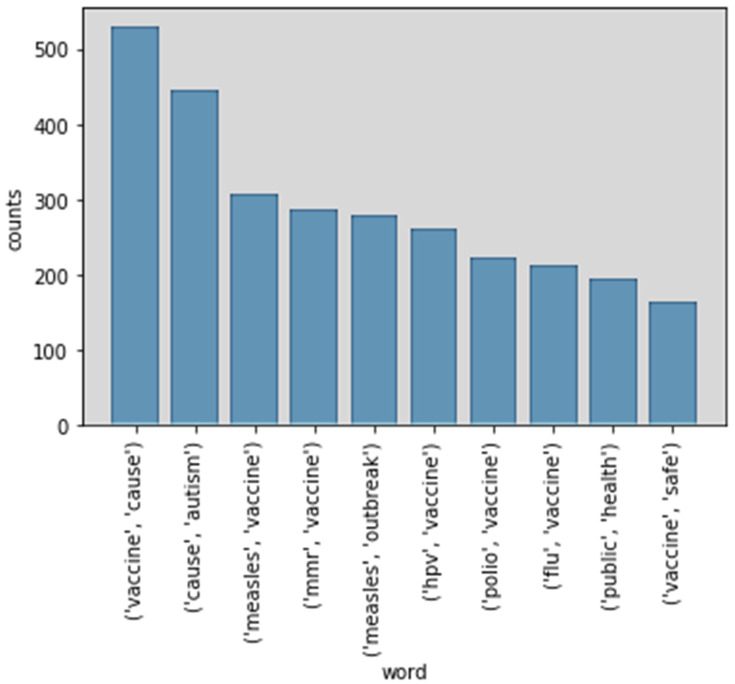
Top 10 bigram phrases.

**Figure 10:**
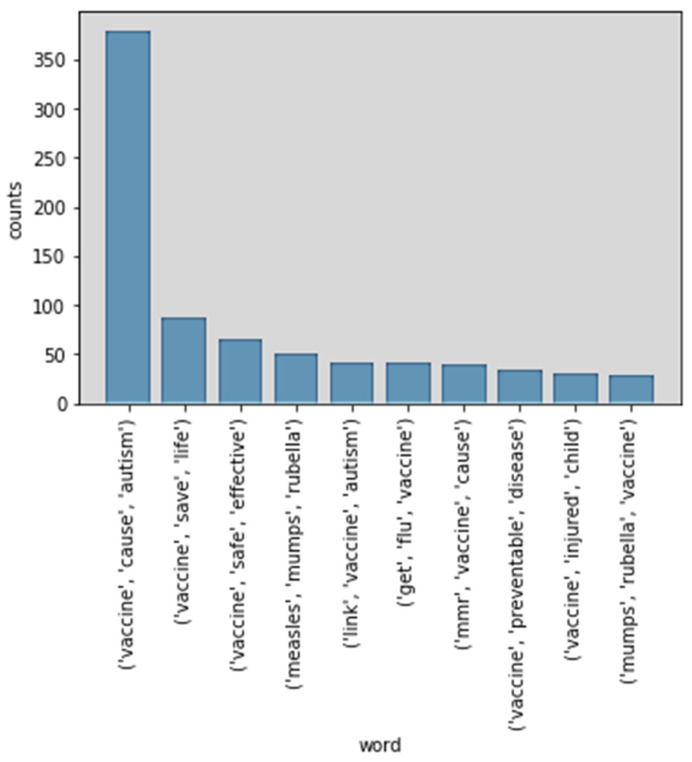
Top 10 trigram phrases.

To understand the relationships between frequency words, the study identified word pairs (bigram and trigram). It counted the number of times a word pair appeared. The bigram and trigram make (statistical) predictions about what is happening in a sentence. They help researchers understand vaccine-related data extracted from Twitter. For example, a particular word may appear or an element belonging to a particular word class may appear. A bigram makes a word prediction based on the prior word. A trigram makes a word prediction based on the two prior words. Figure 9 (bigram) and Figure 10 (trigram) show the top 10 bigram and trigram phrases and counts. The words “vaccine cause” (529), “cause autism” (444) and “measles vaccine” (307) are the top three terms in the bigram phrases.

Figure 10 shows the top 10 trigram phrases in vaccine-related tweets. “Vaccine cause autism” (380), “vaccine save life” (87), and “vaccine safe effective” (66) are the top three trigram phrases. There are three negative phrases among the top 10 trigram phrases. The first, “vaccine cause autism,” is also the most frequent word (380 mentions). The other two negative phrases are “link vaccine autism” (41) and “vaccine injured child” (30). Positive phrases in the top 10 trigram phrases are “vaccine save life” (87), “vaccine safe effective” (66) and “vaccine preventable disease” (34). However, the number of all the positive phrases is less than the number of “vaccine cause autism.”

**Figure 11:**
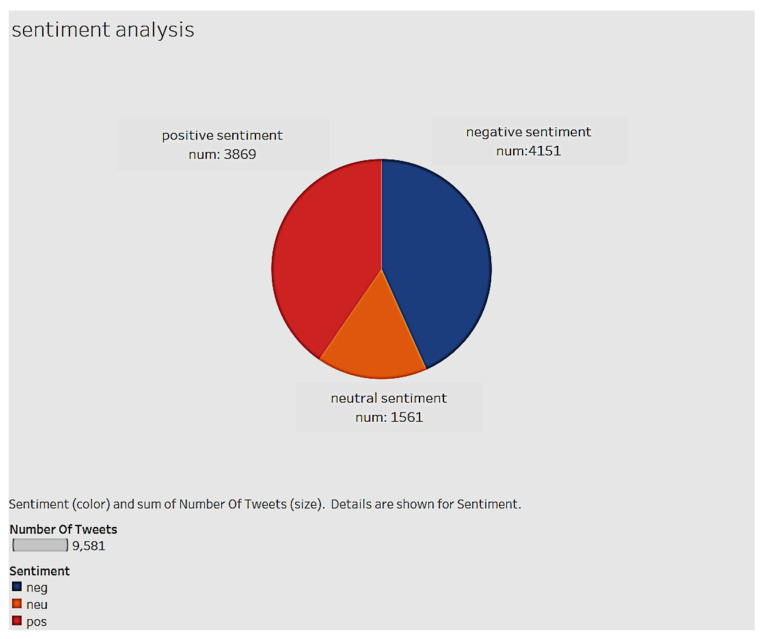
Positive, neutral and negative sentiment tweets in the data set.

Of the 9581 vaccine-related tweets in this dataset, 4151 were classified as negative sentiment tweets, 3869 tweets were positive sentiment tweets and 1561 tweets were neutral sentiment (see Figure 11). According to this pie chart, 43.3% were negative tweets, 40.4% were positive tweets, and 16.3% were neutral tweets. The number of negative tweets was slightly higher than positive tweets.

The TF-IDF technique searches through a corpus of documents to determine which words are favorable for a query. This study transforms the text data into a TF-IDF matrix to provide the frequency of a word in the corpus.

**Figure 12:**
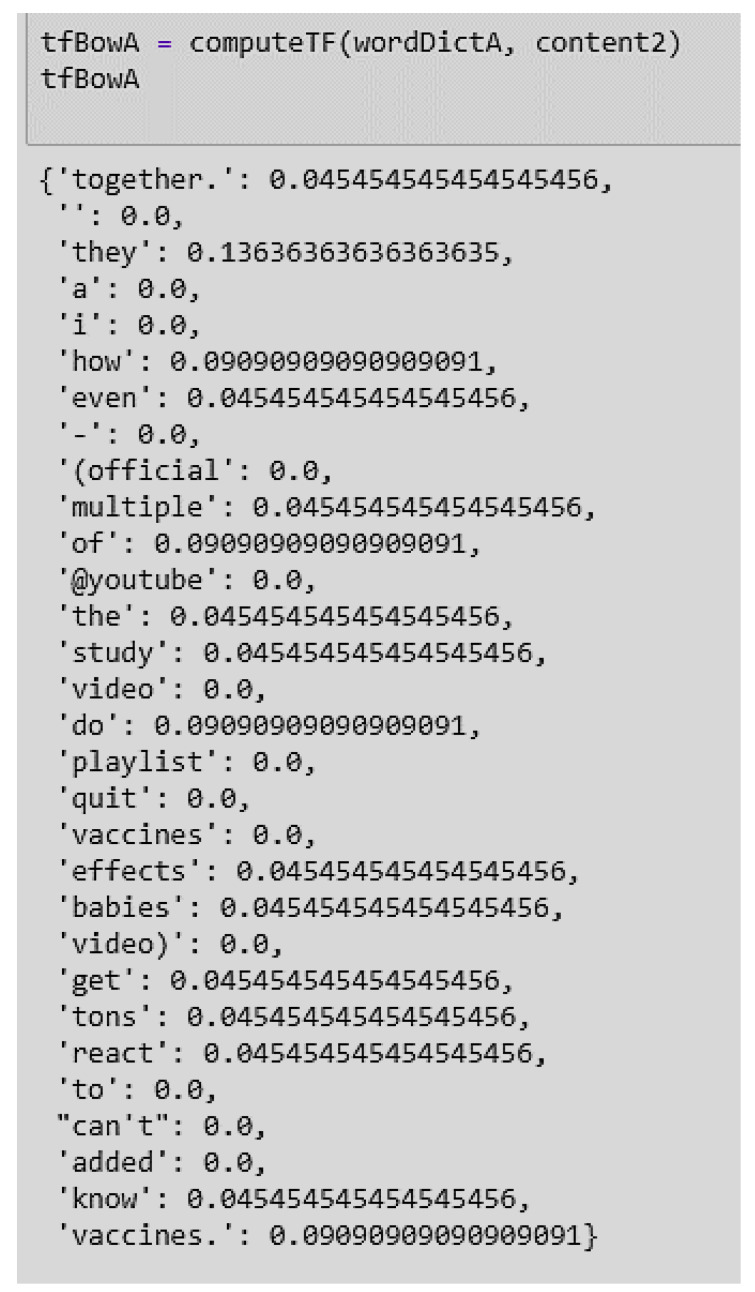
Example of a term frequency score of Document 1 in the dataset.

**Term Frequency (tf):** This provides the frequency of words in each document in the corpus. It is the ratio of the number of times the word appears in a document compared to the total number of words in that document. TF increases as the number of occurrences of the word increases within the document. Each document has its own term frequency (Figure 12).

**Figure 13:**
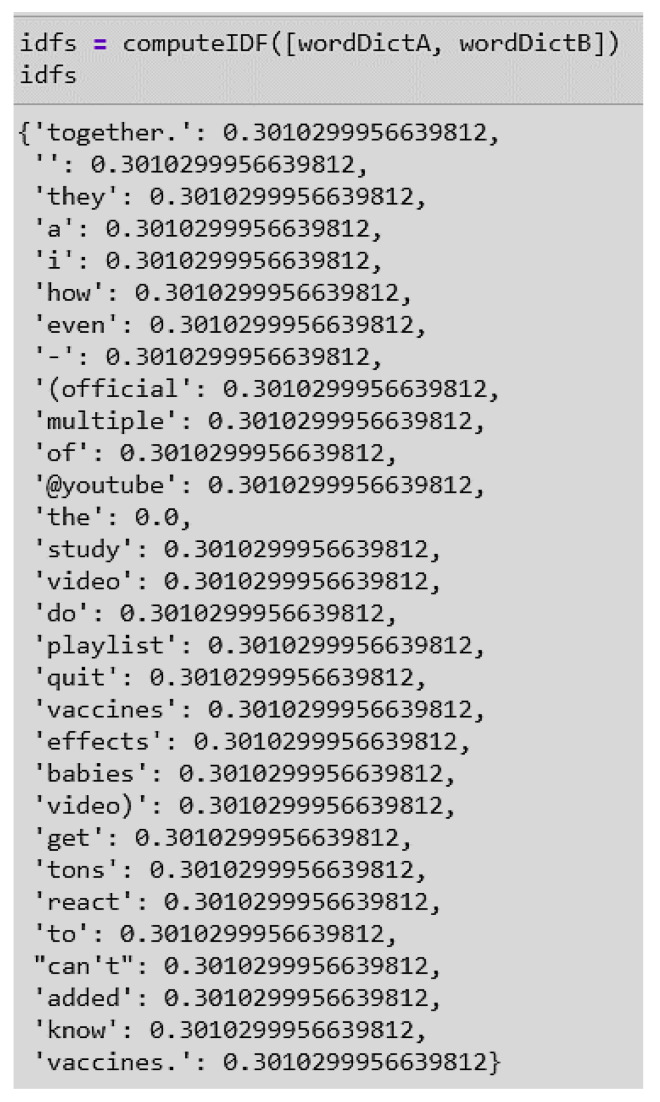
Example of inverse data frequency (IDF) scores in Document 1 and 2 of the data set.

**Inverse Data Frequency (idf):** This measure is a calculation of the weight of rare words across all documents in the corpus. Words that rarely occur in the corpus have a high IDF score (Figure 13).

**Figure 14:**
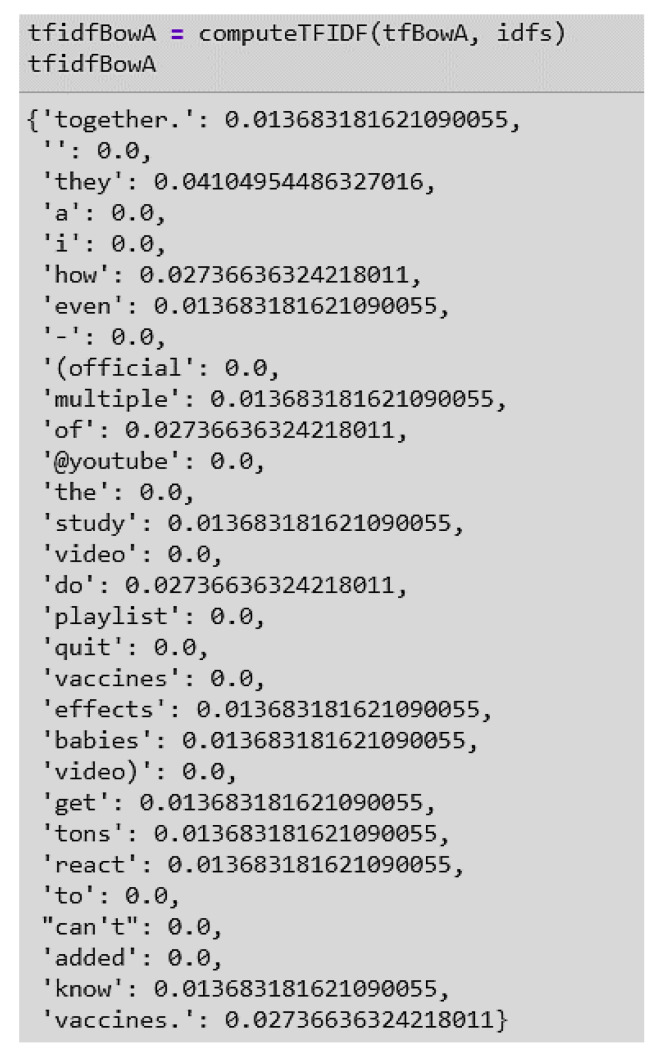
Example of the term frequency–inverse document frequency (TF-IDF) score from Document 1 in the data set.

The product of TF and IDF gives the TF-IDF score for a word in a document in the corpus (Figure 14). A high TF-IDF indicates a strong relationship with the document in which it occurs.

**Figure 15:**
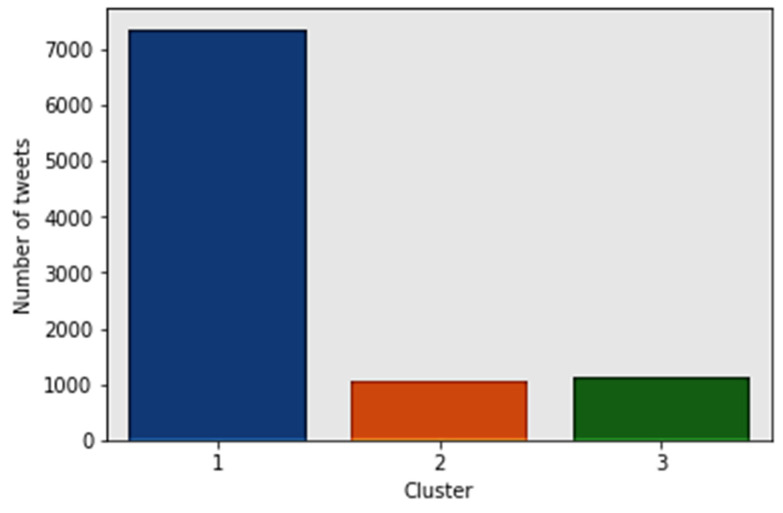
Tweets per cluster.

Using the TF-IDF matrix, clustering algorithms can be run to better understand the hidden structure within a data set. In this study, k-means clustering was used. K-means initializes with a predetermined number of clusters (for example, three). In order to minimize the within-cluster sum of squares, each observation is assigned a cluster (or cluster assignment). Next, the mean of the clustered observations is calculated and used as the new cluster centroid. After this, observations get reassigned to clusters, and centroids get recalculated. This is done iteratively until the algorithm reaches convergence. Figure 15 shows the number of tweets per cluster. Cluster 1 has the greatest number of tweets (7361). Clusters 2 and 3 have 1076 tweets and 1145 tweets, respectively.

**Figure 16:**
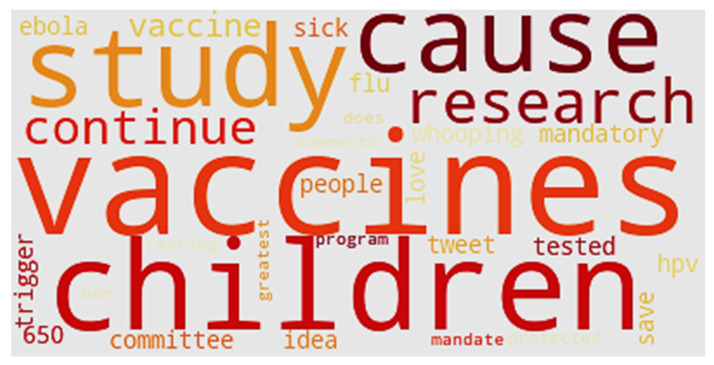
Word cloud of Cluster 1.

Figure 16 shows the word cloud of Cluster 1. Words like “study,” “research,” and “program” show that utterances in Cluster 1 discuss studies or innovations in the field of vaccines. Words like “mandatory” and “mandate” are attitudes surrounding vaccinations. Words such as “Ebola,” “HPV,” and “flu” show the diseases being worked on by researchers. Vaccine breakthroughs related to these diseases will draw the public’s attention. Twitter users in the first group discuss breakthroughs related to vaccines and patients. Tweets in Cluster 1 have the greatest count that do not share debatable information or misinformation about vaccines. Most utterances in the vaccine-related tweets discuss scientific issues.

**Figure 17:**
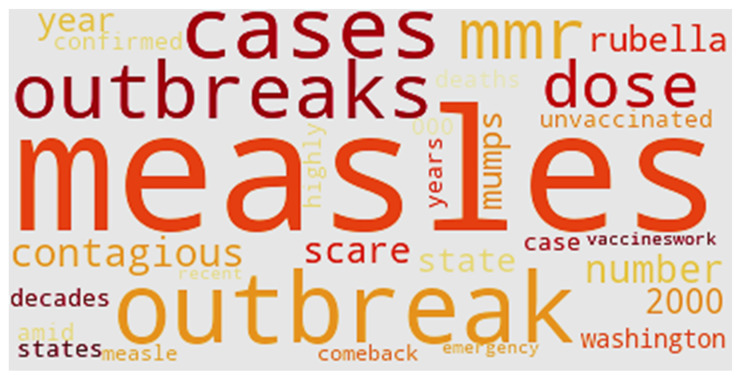
Word cloud of Cluster 2.

Figure 17 shows the word cloud of Cluster 2. “Measles” appears in the middle of the cloud because tweets in this cluster focus on measles. Noticeable words also include “contagious,” “outbreak,” and “comeback.” The tweets in Cluster 2 focus on outbreaks in the U.S. The word “2000” refers to the year measles was declared eliminated in the U.S. “Washington” refers to news surrounding the 50 confirmed cases of measles in the state in February 2019. “Scare” and “emergency” provide a description of public sentiment surrounding the outbreak. The reason for the significant outbreak is also listed in Figure 17—“unvaccinated.”

**Figure 18:**
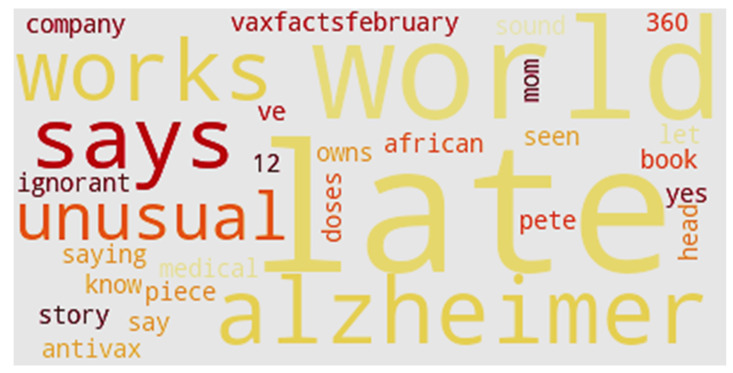
Word cloud of Cluster 3.

**Figure 19:**
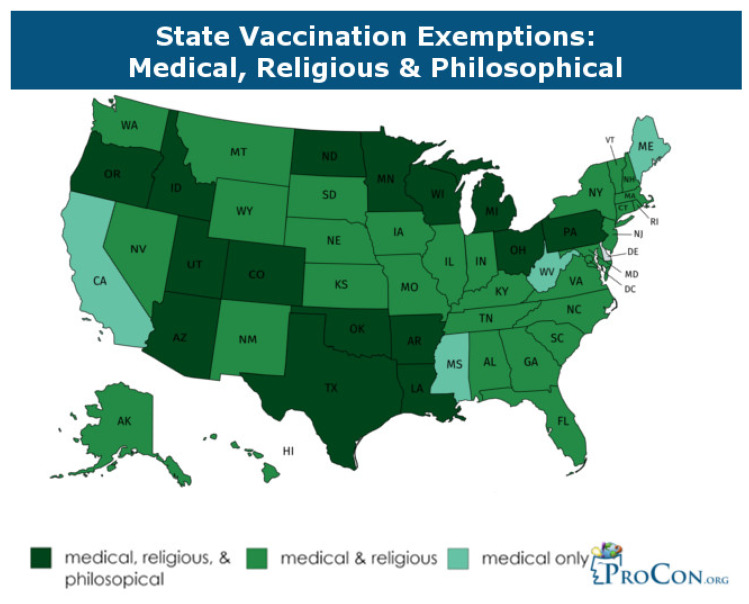
Vaccination exemptions by state (2018).

Figure 18 shows the word cloud of Cluster 3. “Alzheimer” is a noticeable word at the bottom of the cluster. Another interesting word is “Pete,” which represents Pete Buttigieg, a two-term mayor of South Bend, Indiana. After an initial statement that the 2020 Democratic presidential candidate supported some religious and personal exemptions, Buttigieg sought to clarify his position on vaccines. Initially, his statement was criticized by Democratic activists as identifying with antivaccination proponents (antivaxxers) [115]. Only four U.S. states have medical exemptions: West Virginia, California, Maine and Mississippi. California and Maine have only recently approved medical exemptions, while West Virginia and Mississippi have practiced vaccination exemptions based on medical reasons for many years. Other kinds of exemption include religious and philosophical types (Figure 19).

## 5. Discussion

This is one of the few studies to analyze online sentiment, word usage and attitudes on the measles vaccine using text mining and sentiment analysis on social media, specifically Twitter. The focus is on information related to vaccines and measles, which also includes health misinformation. The method can also be applied to other health domains and areas. Findings show that Twitter discussions focus on vaccine-related topics of measles, children, autism and parents, demonstrating public concern in these areas. The number of tweets with negative sentiments was only slightly higher than those with positive or neutral sentiments. The negative sentiments mostly centered on the link between vaccine and autism, the vaccine being a cause for autism, and the vaccine causing injury to children. The positive sentiments related to the existence of a vaccine for measles, the vaccine being effective and the vaccine actually saving lives. In this context, we need to highlight that all the tweets were analyzed, regardless of whether they originated from one or multiple users. The discussions converge in three holistic clusters: discussions on innovations in the arena of vaccines; discussions on outbreaks in the U.S; and frequency discussions on medical exemptions of vaccines by the states in the U.S. This depicts public concern on a range of issues related to disease and disease prevention, thus offering a lens into the level of awareness of public health.

It is interesting to explore factors that can contribute to the online posting of negative sentiments. In an empirical study of Facebook users, it was demonstrated that positive information gets disseminated fast but does not sustain as long as negative information [15,116,117,118]. In this context, future studies can investigate whether there is an optimal period in which information can be presented online to create a positive influence and keep it active in memory. It is also worth studying whether people post negative sentiments on vaccines just as an attention-seeking gesture of offering radically differing opinions. Particularly in healthcare, it is worth looking at a means to motivate people with positive sentiments to remain active and contribute more online. Positive emotions have been suggested to incite people to consider long-term benefits over short-term costs [119,120,121]. Lastly, considering the affinity of users in different age groups to certain platforms, future studies can incorporate hybrid methods involving multiple platforms to be able to compare sentiments across age groups and across platforms.

## 6. Scope and Limitations

This study is not without limitations. First, it is a snapshot in time. Public sentiment may change over time. Second, the sentiments expressed in the tweets may not be truthful and may introduce elements of bias into the data. Third, the subjectivity introduced in the use of various techniques such as stop word, stemming, etc., may impact the results. Long-term research should address generalizability and reproducibility of the research. Fourth, sample size is an issue. Future studies may include larger sample sizes. In the data analysis portion of the research, the study used bigram and trigram to study the data. Using the data set, the study concluded that people talked most about “vaccine cause autism.” However, the study cannot show that every tweet with “vaccine cause autism” is a negative sentiment. The six randomly chosen tweets from the data set showed that half of the tweets mentioned “vaccine cause autism.” However, two of the three tweets criticized the opinion, meaning that they sided with the belief that “vaccine save life.” Based on the generated clusters, the study performed a sentiment analysis with each cluster. The word cloud clusters were not persuasive. Additional statistical analysis may shed more light on this.

Nevertheless, the study surfaces the key reasons for vaccine resistance and its direct association with the spread of measles. Additionally, the study is one of the few to demonstrate the efficacy and usefulness of machine learning and text analytics in conducting rapid studies of large sets of social media data to gain insight into public opinion in the public health and infectious disease arenas.

## 7. Conclusions

Overall, the results in this study have promising implications. First, while social media chatter is more about the association between vaccination and autism (misinformation discussed previously), much of the public discussion is about how vaccination can save lives and is safe and effective. The sentiment is that vaccination prevents disease. We highlight this important revelation. At the same time, we urge scientists and public health policy officials to be on high alert regarding the potential for the rapid spread of misinformation, as well as challenging falsehoods, that tend to get firmly entrenched in the public mind (e.g., autism). Second, considering the current era of social media, it is imperative that stakeholders and government officials the world over—being informed through monitoring social media discussion and sentiment—engage the public aggressively and continually in risk communication and education. Third, we show that there is a positive trend in attitudes towards public health, as revealed in the discussion of the role of overall science and research in regard to vaccination, and the association between measles, contagion and a lack of vaccination. Fourth, as old infectious diseases surface again (e.g., Ebola) and new viruses emerge (e.g., COVID-19), and rapid progress is made in the development of new vaccines, we suggest that policy makers be fully mindful of the fact that health is in the context of society. Therefore, paying attention to what the public thinks can lead to informed decisions. Lastly, the use of advanced technology such as machine learning, natural language processing, sentiment analysis and text analysis can accelerate the maturing process of understanding public opinion in the context of the spread of infectious diseases.
